# MC-UNet: Multimodule Concatenation Based on U-Shape Network for Retinal Blood Vessels Segmentation

**DOI:** 10.1155/2022/9917691

**Published:** 2022-11-03

**Authors:** Jun Li, Ting Zhang, Yi Zhao, Nan Chen, Han Zhou, Hongtao Xu, Zihao Guan, Lanyan Xue, Changcai Yang, Riqing Chen, Lifang Wei

**Affiliations:** ^1^College of Computer and Information, Fujian Agriculture and Forestry University, Fuzhou 350002, China; ^2^Digital Fujian Research Institute of Big Data for Agriculture and Forestry, Fujian Agriculture and Forestry University, Fuzhou 350002, China

## Abstract

Accurate retinal blood vessels segmentation is an important step in the clinical diagnosis of ophthalmic diseases. Many deep learning frameworks have come up for retinal blood vessels segmentation tasks. However, the complex vascular structure and uncertain pathological features make blood vessel segmentation still very challenging. This paper proposes a novel multimodule concatenation via a U-shaped network for retinal vessels segmentation, which is based on atrous convolution and multikernel pooling. The proposed network structure retains three layers of the essential structure of U-Net, in which the atrous convolution combining the multikernel pooling blocks are designed to obtain more contextual information. The spatial attention module is concatenated with the dense atrous convolution module and the multikernel pooling module to form a multimodule concatenation. And different dilation rates are selected by cascading to acquire a larger receptive field in atrous convolution. Adequate comparative experiments are conducted on these public retinal datasets: DRIVE, STARE, and CHASE_DB1. The results show that the proposed method is effective, especially for microvessels. The code will be released at https://github.com/rocklijun/MC-UNet.

## 1. Introduction

The retina is one of the most important parts of the eyes [1]. On the basis of the data published by the WHO, a growing number of people around the world are suffering from eye diseases [2]. The morphological characteristics of retinal blood vessels are very helpful for ophthalmologists who can use morphological features of retinal blood vessels, such as branching patterns, angles, curvatures, widths, and lengths, to diagnose and assess eye diseases [3, 4]. The ophthalmologist can effectively screen and diagnose fundus-related diseases by examining and analyzing the shape structure of retinal blood vessels. Therefore, fundus examination is an important part of the ophthalmic examination. Extracting the shape and structure of retinal blood vessels is the most pivotal procedure in the ophthalmic examination for ophthalmologists to identify diseases. In traditional medical procedures, the retinal vascular area needs to be manually segmented by experienced specialists, which is time-consuming and labor-consuming. Furthermore, the blood vessels in the retinal image are irregular and densely distributed, such as a lot of small blood vessels with low contrast, which is easily confused with the background. Although there are many retinal image segmentation methods that have been presented, those issues make blood vessel segmentation still very challenging.

The unsupervised method and the supervised learning method comprise the retinal vessel segmentation method. The difference between them is whether the input data have manually segmented labels. Oliveira et al. [5] used two algorithms for median ranking and weighted mean, which are different to combine the Frangi filter, matched filter, and Gabor wavelet filter for blood vessels segmentation. Alhussein et al. [6] extracted the enhanced images of thin and thick blood vessels, respectively, based on a hessian matrix and intensity transformation method. Azzopardi et al. [7] presented a selective response vascular filter called B-COSFIRE for vascular segmentation. Saffarzadeh et al. [8] used a multiscale line operator to detect blood vessels and K-means to do blood vessels segmentation. These methods are efficient and fast in retinal vessel segmentation, but the segmentation performances are dependent on the selection of feature extractors. While supervised learning methods can learn features from the original images and segmentation labels that makes it more effective in segmentation tasks owing to get the input-output relationship. And the supervised learning methods can be subdivided into deep learning methods and traditional machine learning methods. The SVM and random forest, which belong to traditional machine learning models, need to manually construct features and map them to the target space. Wang [9] combined the characteristics of Gaussian scale space and the divergence characteristics of a vector field and used the SVM classifier to segment blood vessels. Zhu et al. [10] used Cart and AdaBoost classifiers to classify pixels. Although the traditional machine learning method is easy to understand and can be explained, it requests to fit the feature types and feature selection methods that make the feature representation ability limited.

During the past few years, convolutional neural network (CNN) has made outstanding achievements in the automatic segmentation of retinal vessels. Compared with traditional machine learning, there are many layers of neural networks in deep learning, which has strong nonlinear modelling ability and feature representation ability. In particular, since the U-Net [11] was proposed, various U-shaped networks based on encoding and decoding structures make biomedical images have more accurate segmentation performance. And several excellent retinal vessel segmentation methods that are U-NET based are proposed. Li et al. [12] proposed a method using structural redundancy in the vascular network to find fuzzy vascular details from the segmented vascular images and expand the depth of the model through multiple iterations. Two U-NET-based models, one of which is recurrent and the other is recurrent residual, have been proposed by Alom et al. [13], using the functions of residual network and RCNN. Zhuang [14] proposes a multi-U-Net chain containing multiple encoder-decoder branches. Yuan et al. [15] fused the multilevel attention module with U-NET to obtain the fusion information of low and high levels for alleviating the problem of the network overfitting and obtaining generalization ability. Wang et al. [16] designed a dual-coding U-NET, which has outstanding performance in improving the segmentation capability of vessels in the retina. A spatial attention module is added to the SA-UNet (Spatial Attention U-Net for Retinal Vessel Segmentation) to obtain more features of spatial dimensions by Guo et al. [17]. The IterMiUnet [18] is designed to alleviate the heavy parameterization of U-Net, inspired by Internet [12] and MiUnet [19]. Zhang et al. [20] designed the Bridge-net to learn context-involved and noncontextual features to obtain superior segmentation results.

Although these U-Nets and their improved networks have been used in retinal vessels segmentation so widely, those suffer from many limitations and deficiencies. The encoder-decoder structures receive the information feature and its transmission in the same layer by jump connections, which may cause the loss of small and fragile vessels owing to the limited comprehensive features. In order to alleviate the problems, we propose a multimodule concatenation network based on a U-shape network called MC-UNet for retinal vessel segmentation, which retains local and global information about the retinal main blood vessels and capillaries. Furthermore, the contributions that this paper can make are summarized as follows:

1. We proposed a multikernel pooling based on the U-shape network that retains three layers, the essential structure of U-Net, but the atrous convolution combining the multikernel pooling blocks are designed to obtain more contextual information.
2. We design a multimodule concatenation network to contain local and global information for retaining small vascular and advanced features.
3. The spatial attention module in the network is concatenated with the dense atrous convolution module and multikernel pooling module, which can further enhance the significance of the target.
4. We evaluate and analyze the proposed MC-UNet on the challenging task of retinal blood vessels segmentation. According to the results of experiments, our method reaches the state-of-the-art level on the public datasets.

## 2. Methods

**Figure 1:**
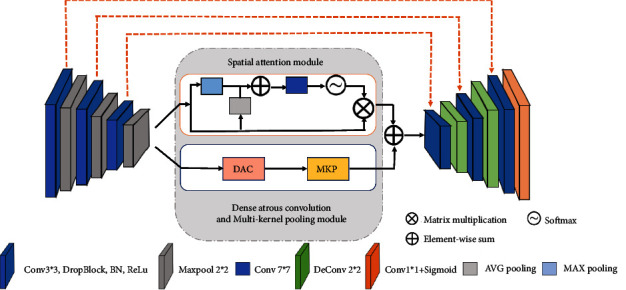
Diagram of the proposed MC-UNet.

In this section, we will introduce our proposed MC-UNet shown in Figure 1. Our network retains three layers, the essential structure of U-Net with a spatial attention module the same as SA-UNet [17]. There are three skip connections and a four layers network structure in our proposed method and is different from the five layers network structure of the original U-NET. The Dropblock and BN modules are used to take the place of the convolution block in the original U-NET, which can effectively prevent overfitting of the network and improve network training speed. Consequently, it is more suitable for small sample data sets. The main improvement for our proposed is to bind the dense atrous convolution module (DAC) and multikernel pooling module (MKP), which joint local and global information for a certain extent. Then, the spatial attention module in the network is concatenated with DAC and MKP. For each layer, it is including a Conv3 ∗ 3, Dropblock, BN modules, ReLu and a 2 ∗ 2 max-pooling. We will elaborate on the MC-UNet in detail in the following subsections.

### 2.1. Spatial Attention Module

**Figure 2:**
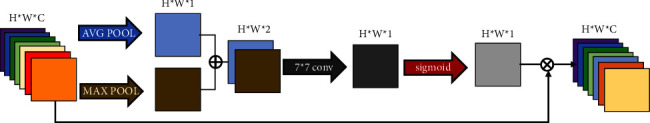
The illustrations of spatial attention module (SA).

The spatial attention module [21] generates a spatial attention map using the maximum pool and average pool operations, selectively paying attention to the feature information in the image and ignoring other background information. The output feature *SA* is obtained by multiplying the input feature *F* and attention map *σ*(*·*), which is shown in the following formula:

(1) $$\begin{array}{c} SA=F∙\sigma \left(f^{7}\left(\left[maxpool\left(F\right);avgpool\left(F\right)\right]\right)\right)\text{,} \end{array}$$

where *f*^7^ and *σ* represent 7 ∗ 7 convolution operation and the sigmoid function, respectively. The illustration of spatial attention module is shown in Figure 2.

### 2.2. Dense Atrous Convolution Module

**Figure 3:**
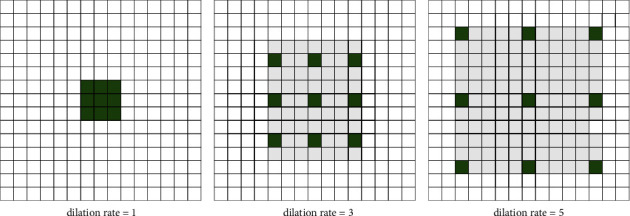
Three atrous convolution of different dilation rate, the dilation rates are 1, 3, and 5, respectively.

Atrous convolution has a widespread application in semantic segmentation, target detection and other tasks by many classical networks, such as DeepLab [22, 23]. In deep learning algorithms, profit from pooling layer and convolution layer, the receptive field of feature image is increased and the size of feature image is reduced. What's more, upsampling is used to make the image size restored. But now, due to the process of feature image shrinkage and magnification, the accuracy will be lost. Atrous convolution can increase the receptive field and maintain the size of the feature map to reduce the computation of the network, which is utilized to replace downsampling and upsampling. The dilation rate of the atrous convolution can be set with different values, by which different receptive fields can be achieved for multiscale information.

(2) $$\begin{array}{c} y\left(i\right)=\underset{k}{\sum }x\left[i+r\times k\right]w\left[k\right]\text{,}k=1,2,\cdots ,k, \end{array}$$

where *r* represents the dilation rate and *k* is the length of the filter *w*. In particular, when *r* = 1, formula (2) is the standard convolution. The input feature maps *x* are convolved with a filter *w* to obtain the output *y*. And Figure 3 shows the schematic diagram of the atrous convolution, the dilation rates are 1, 3, and 5, respectively. And the small dilation rates can obtain the local information and the big ones can get the global information that makes the network extract local and global information for retaining small vascular and advanced features.

**Figure 4:**
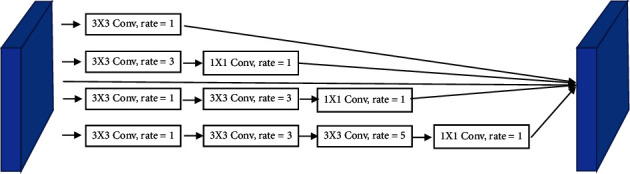
The illustrations of dense atrous convolution (DAC).

Compared with downsampling, atrous convolution can both enlarge the receptive field nicely and accurately locate the target and reduce the loss of spatial resolution. The dense atrous convolution [24] module shown in Figure 4 is generated by integrating the atrous convolution using different dilation rates, which can capture the context information of different scales and achieve local or global information. By using different dilation rates *r*_*k*_ to combine the atrous convolution, the output *D* of atrous convolution modules can be obtained.

(3) $$\begin{array}{c} D=\underset{k}{\sum }y_{r_{k}}\left(x\right)\text{.} \end{array}$$

### 2.3. Multikernel Pooling Module

**Figure 5:**
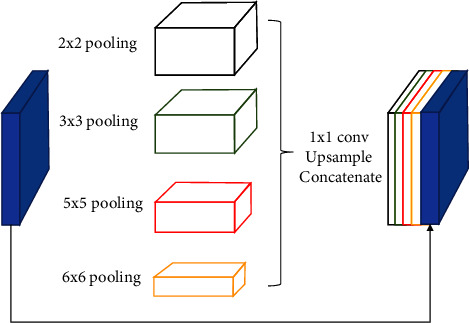
The illustrations of residual multikernel pooling.

The multikernel pooling [24] module is changed based on the spatial pyramid [25], which can make the redundant information of the feature map be reduced and the amount of calculation. According to the different sizes of the kernel, the feature information of receptive fields with different sizes is extracted to increase the segmentation performance of the model. The multikernel pooling module is introduced into the SA-UNet, which relies on multiple different kernels to detect different sizes targets. Multikernel pooling can use more context information by combining general max-pooling operations of different kernel sizes, as shown in Figure 5. And encoding the global context information into four receiving domains of different sizes: 2 × 2, 3 × 3, 5 × 5, and 6 × 6. Then, a 1 × 1 convolution is carried out to reduce the dimension of feature mapping, and upsampling is carried out to get features of the same size as the original feature mapping. Lastly, we concatenate the original features and the upsampled feature mapping and obtain the output feature *MKP* of multikernel pooling module.

(4) $$\begin{array}{c} MKP=\underset{i}{\sum }f^{1}\left({Maxpool}_{k_{i}}\left(D\right)\right)\text{,} \end{array}$$

where *f*^1^ and *k*_*i*_ denote the 1 × 1 convolution and *i*_*th*_ kernel of different sizes, and the *D* is the output feature map representing the dense atrous convolution module.

The encoder-decoder structures receive the information feature and its transmission in the same layer by jump connections, which may cause the loss of small and fragile vessels owing to the limited comprehensive features. The spatial attention module, multikernel pooling module and dense atrous convolution module are complementary in the ability and scope of feature acquisition. Inspired by them, we propose a multimodule concatenation network for accurate retinal vessel segmentation. The output feature map *F* is obtained by concatenating the output features of the spatial attention module *SA* and the multikernel pooling module *MKP*.

(5) $$\begin{array}{c} F=SA+MKP\text{.} \end{array}$$

## 3. Experiments

### 3.1. Datasets

We use the fundus datasets which are publicly available to verify our method: DRIVE [26] (digital retinal images for vessel extraction), CHASE_DB1 [27] (child heart and health study in England), and STARE [28] (structured analysis of the retina) to evaluate the segmentation performance of our approach MC-UNet. The STARE dataset includes pathological abnormal and healthy retinal images, which can be used to evaluate the impact of the model on abnormal fundus images. The specific information of the three datasets is shown in Table 1.

### 3.2. Evaluation Criteria

The aim of retinal vascular binary classification work is to divide each pixel in the input images into two categories: vascular (positive) and background (negative). By comparing the segmentation maps with the true value of the label, four indexes can be obtained: TP, TN, FP, and FN. P represent the number of white pixels in true images; N represents the number of black pixels in the true image; T for true; and F for false. TP represents the number of white pixels correctly predicted by optic disc, while TN represents the number of black pixels correctly predicted by the optic disc. The values of TP, TN, FP, and FN are calculated according to the total number of pixels in the ground-truth images.

On the basis of these four basic indexes, accuracy (ACC), sensitivity (SEN), specificity (SP), area enclosed by the coordinate axis under the ROC curve (AUC), and F1-score can be calculated [17]. In our experiment, almost all the above indicators are used. The calculation formulas are as describe as follows:

(6) $$\begin{array}{c} ACC=\frac{TP+TN}{TN+FP+TP+FN}\text{,}\\ SE=\frac{TP}{TP+FN}\text{,}\\ SP=\frac{TN}{TN+FP}\text{.} \end{array}$$

## 4. Results

**Figure 6:**
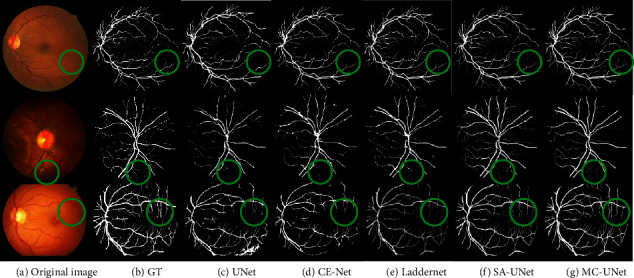
The segmentation example of the three datasets. Among them, (a) is the original retinal image, and (b) is the ground truth. From (c) to (g) are segmentation maps by U-Net, CE-Net, LadderNet, SA-UNet, and our method, respectively. The DRIVE dataset is shown in the first row, the CHASE_DB1 dataset is shown in the second row, and the STARE dataset is shown in the last row.

On the three datasets, we train and evaluate our method by using the manual annotation marked by the first expert. The segmentation result examples from the DRIVE, STARE, and CHASE_DB1 datasets are shown in Figure 6, which perceive the comparisons of the segmentation results on the three datasets with other methods are listed, including some methods based on U-Net. From Figures 6(a)–6(g), there are the original color retinal image, the ground truth, the segmentation map by U-Net [11], CE-Net [24], LadderNet [14], SA-UNet [17] and proposed method, respectively. Furthermore, all the experiments were carried out on NVIDIA Quadro M5000 and 3.00 GHz PCs. It can be observed in Figure 6 that the proposed MC-UNet achieves better performance than others, obtaining more vessels in a representative patch (green disc) in the vascular tree terminal region regions.

We also compare the segmentation results on the three datasets with other methods by the five evaluation criteria shown in Table 2. Notably, MC-UNet achieves the best performance on DRIVE and CHASE_DB1. And by comparing with the backbone, our method has better performance, which illustrates that the proposed framework is effective for vascular segmentation. Specifically, the SE and AUC of our framework on three datasets are higher than backbone SA-UNet. Our method has the highest ACC, SP, and AUC on DRIVE, the highest ACC, SE, and AUC on CHASE_DB1. Due to many lesion images in the STARE dataset, the sensitivity index is not satisfactory by MC-UNet. However, compared with the backbone network, the proposed MC-UNet obtains better performances which also verify our method is effective.

**Figure 7:**
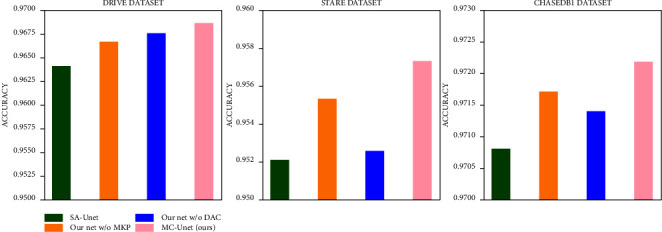
The accuracy indicator histogram for justification of the MKP module and DAC module. The green bar represents the ACC results segmented by SA-UNet, the orange bar represents the ACC results by our proposed framework without MKP module, the blue bar represents the ACC results by our proposed framework without DAC module and the pink bar represents the ACC results segmented by our proposed framework MC-UNet with MKP module and DAC module.

**Figure 8:**
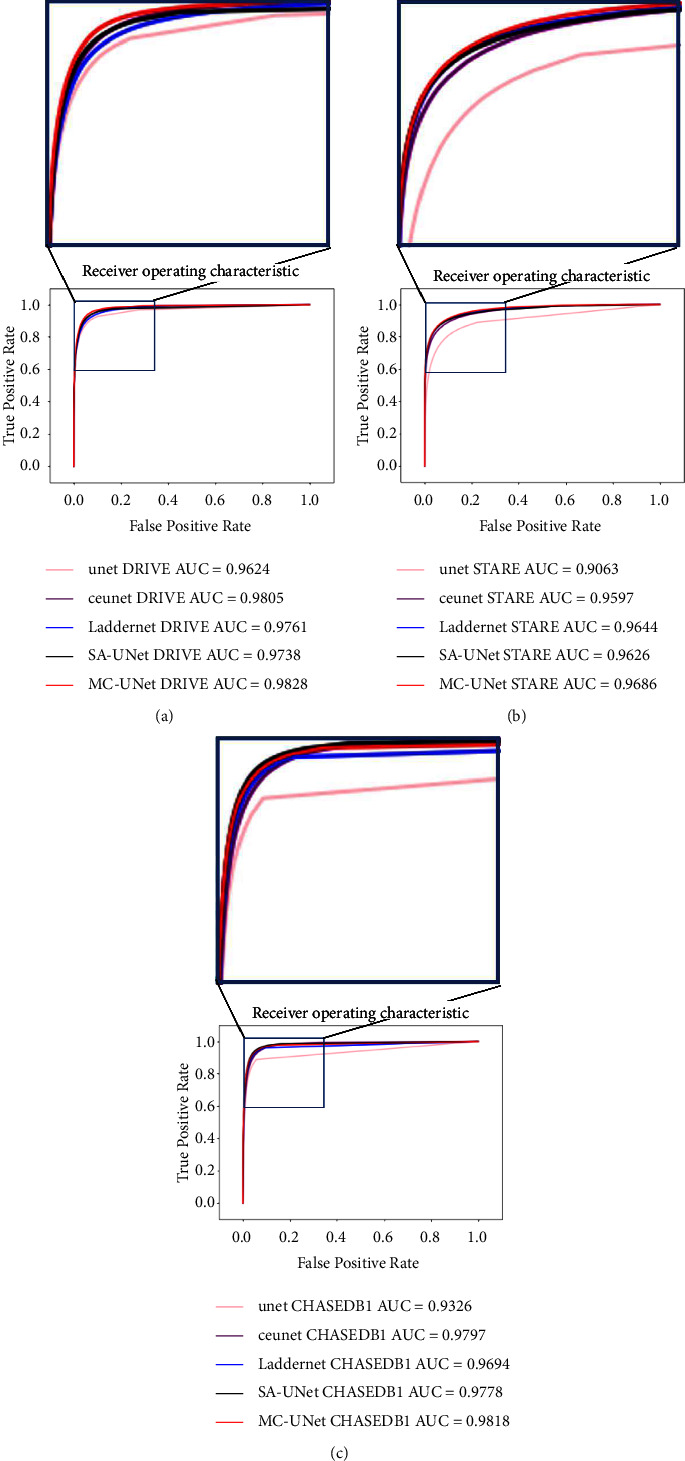
The ROC curves of different method on DRIVE (a), STARE (b) and CHASE_DB1 (c) datasets.

Table 3 shows the ablation experiments of the proposed model, where the proposed MC-UNet is compared with the backbone network (SA-UNet), SA-UNet + DAC, and SA-UNet + MKP. It is observed that the DAC module is able to enhance the specificity of the image effectively, reduce the blood vessels rate of false positive in the fundus image, and reduce the misdiagnosis cost of fundus image samples. The MKP module improves the AUC of the segmentation algorithm, making the algorithm more robust. Integrating the DAC and MKP modules into SA-UNet improves the segmentation effect as a whole, reduces the misdiagnosis rate of the image, and improves the ability to predict blood vessels by the algorithm.  Figure 7 more intuitively shows the change of ACC in the ablation experiment.  Figure 8 compares the ROC curves of five different methods on three datasets. It can be seen from the results that our method achieves the best effect.

And Table 4 shows the comparison on parameters for justification of the MKP module and DAC module, which shows that our method has much fewer parameters than the 7.76 M parameters of original U-Net.

## 5. Discussions

There are three skip connections and four layers in our proposed method, compared with four skip connections and five layers in the original U-Net. Although our network has added multiple integrated modules, it has a much smaller number of parameters compared with the original U-Net with 23 convolutional layers and is a lightweight network. The proposed network can enhance the specificity of the image effectively and reduce the blood vessels rate of false positives in the fundus image by integrating the DAC and MKP modules into SA-UNet. However, the limited images available in the dataset restrict the performance of the algorithm. In the experiment, we set a certain number of iterations to avoid overfitting. And we only consider the solution of the same data domain. The domain adaptation method can be introduced to solve domain shift for cross-training and verification for the robustness of the algorithm.

## 6. Conclusions

In order to solve the limited comprehensive features extracted by the encoder-decoder structure in the U-shaped network, which may lead to the segmentation loss of small fragile capillaries, a novel U-shape network is proposed named multimodule concatenation U-Net (MC-UNet) based on atrous convolution and multikernel pooling for retinal vessels segmentation. The network retains local and global information about the main retinal vessels and capillaries. The DAC and MKP modules are introduced to increase the receptive field for improving the sensitivity of the algorithm and retain more detailed feature information for improving the accuracy of retinal vascular segmentation. Experimental results prove the effectiveness of the method, especially for microvessels. However, for more severe lesions in image data, a robust framework is still needed to be studied and discussed.

## Figures and Tables

**Table 1:** The details of the three datasets of DRIVE, CHASE_DB1, and STARE.

Dataset	Resolution	Numbers of images	Train/test split
DRIVE	565 × 584	40	1 : 1
CHASE_DB1	999 × 960	28	1 : 1
STARE	700 × 605	20	1 : 1

**Table 2:** The comparison of our model and other methods in DRIVE, STARE, and CHASE_DB1.

Dataset	Method	Year	ACC	SEN	SP	AUC	F1
DRIVE	U-Net [11]	2015	96.60	76.82	98.53	97.07	—
	R2U-Net [13]	2018	95.56	77.92	98.13	97.84	—
	LadderNet [14]	2018	95.61	78.56	98.10	97.93	82.02
	IterNet [12]	2019	95.74	77.91	98.17	98.16	82.18
	CE-Net [24]	2019	95.50	79.03	97.69	97.80	—
	SA-UNet [17]	2020	96.41	**81.12**	97.67	97.38	80.27
	AACA-MLA-D-UNet [15]	2021	95.81	80.46	98.05	98.27	**83.03**
	IterMiUnet [18]	2022	95.68	80.53	97.89	98.10	—
	Bridge-net [20]	2022	95.65	78.53	98.18	**98.34**	82.03
	MC-UNet	2022	**96.78**	81.00	**98.79**	98.28	81.49

STARE	U-Net [11]	2015	96.43	77.64	98.65	90.63	—
	R2U-Net [13]	2018	97.12	**82.98**	98.62	**99.14**	—
	LadderNet [14]	2018	96.13	78.22	98.04	96.44	79.94
	IterNet [12]	2019	**97.60**	79.69	98.23	98.37	80.73
	CE-Net [24]	2019	97.32	79.09	97.21	95.97	—
	SA-UNet [17]	2020	95.21	71.20	99.30	96.26	77.36
	AACA-MLA-D-UNet [15]	2021	96.65	79.14	98.70	98.64	82.76
	IterMiUnet [18]	2022	96.49	80.69	98.31	98.52	—
	Bridge-net [20]	2022	96.68	80.02	98.64	99.01	**82.89**
	MC-UNet	2022	95.72	73.60	**99.47**	96.86	78.65

CHASE_DB1	U-Net [11]	2015	96.43	77.64	**98.65**	93.26	—
	R2U-Net [13]	2018	96.34	77.56	98.20	98.15	—
	LadderNet [14]	2018	96.56	79.78	98.18	96.94	80.31
	IterNet [12]	2019	97.02	79.69	98.23	98.13	80.73
	CE-Net [24]	2019	96.33	80.08	97.23	97.97	—
	SA-UNet [17]	2020	97.08	81.51	98.09	97.78	77.36
	AACA-MLA-D-UNet [15]	2021	96.73	84.02	98.01	98.74	82.48
	IterMiUnet [18]	2022	95.91	**84.43**	97.04	98.12	—
	Bridge-net [20]	2022	96.67	81.32	98.40	**98.93**	**82.93**
	MC-UNet	2022	**97.14**	83.66	98.29	98.18	77.41

**Table 3:** The ablation experiment results (%) of vessel segmentation on DRIVE, CHASE_DB1, and STARE dataset for justification of the MKP module and DAC module.

		DRIVE			CHASE_DB1			STARE		
DAC	MKP	ACC	SP	AUC	ACC	SP	AUC	ACC	SP	AUC
		96.41	97.67	97.38	97.08	98.09	97.78	95.21	99.30	96.26
√		96.67	98.90	97.96	97.17	98.22	98.17	95.28	98.93	95.83
	√	96.72	98.29	98.42	97.22	98.10	98.29	95.58	99.23	96.50
√	√	96.78	98.79	98.28	97.14	98.29	98.18	95.72	99.47	96.86

**Table 4:** The comparison on parameters for justification of the MKP module and DAC module.

DAC	MKP	Parameters (M)
		0.54
√		2.36
	√	0.69
√	√	2.37

## Data Availability

The datasets used and analyzed during the current study are available from the corresponding author on reasonable request.
